# Genetic characterization of inbred lines of Chinese cabbage by DNA markers; towards the application of DNA markers to breeding of F_1_ hybrid cultivars

**DOI:** 10.1016/j.dib.2015.11.058

**Published:** 2015-12-11

**Authors:** Kazutaka Kawamura, Takahiro Kawanabe, Motoki Shimizu, Keiichi Okazaki, Makoto Kaji, Elizabeth S. Dennis, Kenji Osabe, Ryo Fujimoto

**Affiliations:** a Graduate School of Science and Technology, Niigata University, Ikarashi-ninocho, Niigata 950-2181, Japan; b Graduate School of Agricultural Science, Kobe University, Rokkodai, Nada-ku, Kobe 657-8501, Japan; c Watanabe seed Co., Ltd., Machiyashiki, Misato-cho, Miyagi 987-0003, Japan; d CSIRO Agriculture, Canberra, ACT 2601, Australia; e Plant Epigenetics Unit, Okinawa Institute of Science and Technology Graduate University, Onna-son, Okinawa 904-0495, Japan; f JST PRESTO, Honcho 4-1-8, Kawaguchi, Saitama 332-0012, Japan

**Keywords:** Brassica rapa, F_1_ hybrid cultivar, Marker assisted selection, Fusarium yellows, Clubroot disease, Self-incompatibility

## Abstract

Chinese cabbage (*Brassica rapa* L. var. *pekinensis*) is an important vegetable in Asia, and most Japanese commercial cultivars of Chinese cabbage use an F_1_ hybrid seed production system. Self-incompatibility is successfully used for the production of F_1_ hybrid seeds in *B. rapa* vegetables to avoid contamination by non-hybrid seeds, and the strength of self-incompatibility is important for harvesting a highly pure F_1_ seeds. Prediction of agronomically important traits such as disease resistance based on DNA markers is useful. In this dataset, we identified the *S* haplotypes by DNA markers and evaluated the strength of self-incompatibility in Chinese cabbage inbred lines. The data described the predicted disease resistance to Fusarium yellows or clubroot in 22 Chinese cabbage inbred lines using gene associated or gene linked DNA markers.

**Specifications Table**

**Table:** 

Subject area	Biology
More specific subject area	Plant molecular biology
Type of data	Table, figure
How data was acquired	PCR, Pollination test
Data format	Raw and analyzed data
Experimental factors	Inbred lines of Chinese cabbage used in Kawamura et al. [1] were used for plant materials. DNA isolated from leaves was used as PCR templates.
Experimental features	Gene associated or linked DNA markers were tested to predict the disease resistance. The strength of self-incompatibility was calculated by the number of seeds per flower.
Data source location	Kobe, Japan
Data accessibility	The data is available with this article.

**Value of the data**

• Prediction of disease resistance by DNA markers is useful for marker-assisted breeding.
• Identification of the *S* haplotype is important for determining suitable combinations of parental lines in Brassica vegetables.
• The strength of self-incompatibility is an important factor for F_1_ seed production to avoid inbreeding seed contamination in Brassica vegetables.

## 1 Data

### 1.1 Prediction of Fusarium yellows resistance by DNA markers

Fusarium yellows is caused by a soil-borne fungus *Fusarium oxysporum* f. sp. *conglutinans*/*F. oxysporum* f. sp. *rapae*. Plants infected with Fusarium yellows show leaf yellowing, wilting, defoliation, stunted growth, and death of the host plant, and resistance genes have been identified in *Brassica rapa* [2], [3].

We developed 22 inbred lines of Chinese cabbage as candidates for parental lines of F_1_ hybrid cultivars, especially as seed parents, and the genetic relationship of these 22 inbred lines was evaluated [1]. We have developed dominant DNA markers, Bra012688m and Bra012689m, which are closely linked to the Fusarium yellows resistance locus [3]. Both PCR based and inoculation tests have previously been performed in 7 of the 22 inbred lines using these markers (Table 1) [3], and we assessed these 2 DNA markers against the remaining 15 inbred lines. Twelve of the 15 inbred lines showed PCR amplification of both DNA markers (Table 1), suggesting that these inbred lines have Fusarium yellows resistance.

### 1.2 Prediction of clubroot disease resistance by DNA markers

Clubroot disease is caused by a soil-borne obligate plant pathogen *Plasmodiophora brassicae*. Infected plants of clubroot show inhibition of root development by the formation of clubs [4], [5]. Eight clubroot resistance loci (*CRa*, *CRb*, *CRc*, *CRk*, *Crr1*, *Crr2*, *Crr3*, and *Crr4*) were identified in *B. rapa*, and these loci show different responses to the variable isolates of *P. brassicae* [4], [5], [6], [7], [8], [9].

We tested the reported DNA markers located within or linked to the clubroot resistance loci. We used the dominant DNA marker sets, CRaim-T and craim-Q, which were reported to be linked to the clubroot resistance locus, *CRa* [4]. Amplification of the CRaim-T and craim-Q show the resistant and susceptible genotypes of clubroot disease, respectively [4]. Of 22 inbred lines, 9 showed PCR amplification of CRaim-T (resistant genotype), and 8 of craim-Q (susceptible genotype) (Table 2). No PCR amplification of either primer set was detected in RJKB-T03, -T06, -T08, -T09, and -T11 (Table 2).

We developed a DNA marker, mCrr1a-F/R, by the comparison of sequences between the clubroot resistance gene, *Crr1a,* of resistant and susceptible lines [5]. A susceptible line, A9709, has three large insertions, a 357-bp insertion 37 bp downstream of the start codon, and 333- and 4982-bp insertions in exon 4 [5]. We made a DNA marker in exon 4 of *Crr1a* gene that includes the 333-bp insertion of exon 4 in A9709; larger and smaller amplification fragments are linked to susceptibility and resistance to clubroot disease, respectively. We confirmed that a susceptible line of Chiifu had a larger band. Four inbred lines showed the smaller (resistant genotype) and 14 the larger size band (susceptible genotype), and 4 inbred lines showed no amplification (Table 2).

We used a DNA marker, OPC11-2S, which is linked to the *Crr3* locus [10]. This primer set showed 2 fragment sizes, and the larger band is linked to resistance [10]. Of 22 inbred lines, only RJKB-T16 showed amplification of the larger fragment (resistant genotype) (Table 2).

The dominant DNA marker (TCR108), which is linked to the *CRb*^*Zhang*^ locus, showed that PCR amplification occurs in the resistant genotype [12]. We assayed this DNA marker on 22 inbred lines, of which 15 inbred lines showed PCR amplification (resistant genotype) (Table 2).

The DNA marker (B0902) is linked to the *CRb*^*Kato*^ locus, and amplifies 2 fragment sizes; larger and smaller sized bands are linked to the susceptible and resistant genotypes, respectively [11]. We assayed this DNA marker on 22 inbred lines. Thirteen inbred lines showed PCR amplification producing the larger band (susceptible genotype) and 9 the smaller band (resistant genotype) (Table 2).

Dominant DNA marker sets, B50-C9-FW/B50-RV (resistant genotype) and B50-6R-FW/B50-RV (susceptible genotype), are linked to the clubroot resistance locus, *CRc* [13]. Of 22 inbred lines, no inbred line showed PCR amplification in B50-C9-FW/B50-RV, and 20 inbred lines in B50-6R-FW/B50-RV. No PCR amplification of either primer set was detected in RJKB-T09 and -T14 (Table 2). Of 22 inbred lines, no inbred line showed a resistant genotype.

### 1.3 Identification of *S* haplotypes and evaluation of strength of self-incompatibility

Self-incompatibility, which prevents self-fertilization, is sporophytically controlled by a single multiallelic locus (*S* locus) in Brassica. The determinants of the self-recognition specificity in Brassica are SRK (*S* receptor kinase) in the stigma and SP11 (*S* locus protein 11) in the pollen, both of which are encoded by the *S* locus [14]. Self-incompatibility is successfully used for the production of F_1_ hybrid seeds in *B. rapa* vegetables to avoid non-hybrid seeds, and the strength of self-incompatibility is important for harvesting highly pure F_1_ seeds. As individual plants having the same *S* haplotypes (*S* specific recognition specificity) are incompatible, the *S* haplotypes of parental candidate lines need to be determined. *S* haplotypes are categorized into two classes, class-I and class-II, by sequence homology [14]. The *S* haplotype can be identified by a DNA marker based method, i.e., PCR-RFLP analysis of *SLG (S locus glycoprotein),* which is linked to the *S* locus or dot-blot analysis using *SP11* [15], [16].

**Fig. 1:**
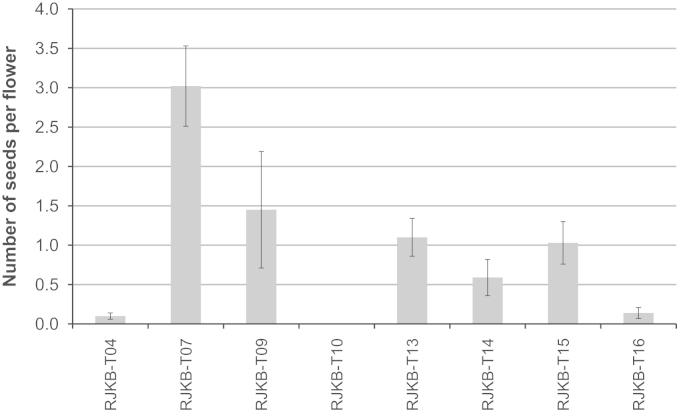
Strength of self-incompatibility evaluated by number of seeds per flower in inbred lines showing *S-40* haplotype.

The *S* haplotypes of the inbred lines were determined by PCR-RFLP analysis [15] or sequencing of the *SLG* gene. In the 22 inbred lines, four class I *S* haplotypes, *S-25*, *S-46*, *S-54* and *S-99,* and two class II *S* haplotypes, *S-40* and *S-60*, were identified (Table 3). To evaluate the strength of self-incompatibility, an artificial self-pollination test was carried out. The average number of seed per flower in inbred lines ranged from 0.00 to 3.02, and RJKB-T04, -T10, -T12, and -T16 had strict self-incompatibility (Table 3). The strength of self-incompatibility varied among inbred lines having the same *S* haplotypes, e.g., *S-40* (Fig. 1).

## 2 Experimental design, materials and methods

### 2.1 Plant materials and DNA extraction

Twenty-two Chinese cabbage inbred lines (RJKB-T01-T20, -T22, and -T24) were used as plant materials [1]. Seeds were sown on soil and plants were grown in growth chambers under a 16-h/8-h light/dark cycle at 22 °C. Leaves harvested from the 2 week seedlings were used for genomic DNA extraction. Total genomic DNA was isolated by the Cetyl trimethyl ammonium bromide method [17].

### 2.2 Identification of *S* haplotypes

Class-I and class-II *SLG* specific primer pairs, PS5+PS15 and PS3+PS21, were used, respectively (Table 4) [15]. The *S* haplotype was identified by PCR-RFLP analysis [15] or direct sequencing of *SLG*. The PCR reaction was performed using the following conditions; 1 cycle of 94 °C for 3 min, 35 cycles of 94 °C for 30 s, 58 °C for 30 s, and 72 °C for 1 min, and final extension at 72 °C for 3 min. For PCR-RFLP analysis, amplified DNA digested by *Mbo* I restriction enzyme was electrophoresed on 13% polyacrylamide gel. The gel was stained with Gelstar solution (0.1 μl/10 ml; Takara Bio). For sequence analysis, the amplified PCR fragments treated by illustra ExoProStar (GE Healthcare) were directly sequenced using ABI Prism 3130 (Applied Biosystems).

### 2.3 Prediction of disease resistance using DNA markers

DNA markers were used to predict the disease resistance in inbred lines. To predict the Fusarium yellows resistance, Bra012688m-F+-R and Bra012689m-F+-R, which were designed from candidate genes for Fusarium yellows resistance, were used [3]. Known resistance gene or linked DNA markers were used to assess the clubroot resistance (*CRa*, CRaim-T-FW+-RV and craim-Q-FW1+-RV [4]; *Crr3*, OPC11-2S-F+-R [10]; *Crr1a*, mCrr1a-F+-R (developed in this study); *CRb*^*Zhang*^, TCR108-F+-R [12]; *CRb*^*Kato*^, B0902-F+-R [11]; *CRc*, B50-C9-FW+B50-RV and B50-6R-FW+B50-RV [13]). The PCR reaction was performed by the following condition; 1 cycle of 94 °C for 3 min, 35 cycles of 94 °C for 30 s, 58 °C for 30 s, and 72 °C for 1 min, and final extension at 72 °C for 3 min. The PCR products were electrophoresed on 1.0% agarose gel. Primer sequences used in this study are shown in Table 4.

### 2.4 Evaluation of strength of self-incompatibility

Seeds were sown on the cell tray, seedlings were transferred into pots two weeks later, and plants were grown in the greenhouse. The strength of self-incompatibility of inbred lines was evaluated in the spring of 2012 by an artificial self-pollination test. The artificial self-pollination test was carried out on 15 flowers of a branch, and 4 or 5 branches from each plant were tested. After flowering, we counted the numbers of pollinated flowers and seeds to calculate the number of seeds per flower (number of seeds/ number of crossing flowers). Lower values for the number of seeds per flower indicate higher strength of self-incompatibility.

## Conflict of interest

The authors declare that they have no conflict of interest.

## Figures and Tables

**Table 1:** Genotype of alleles of Fusarium yellows.

Lines	Marker		Estimation of *YR* allele
	Bra012688m	Bra012689m
RJKB-T01	+	+	R
RJKB-T02	−	−	S
RJKB-T03	+	+	R
RJKB-T04	+	+	R⁎
RJKB-T05	−	−	S
RJKB-T06	+	+	R
RJKB-T07	−	−	S
RJKB-T08	+	+	R
RJKB-T09	+	+	R
RJKB-T10	+	+	R
RJKB-T11	+	+	R
RJKB-T12	+	+	R
RJKB-T13	−	−	S⁎
RJKB-T14	−	−	S⁎
RJKB-T15	+	+	R⁎
RJKB-T16	+	+	R
RJKB-T17	+	+	R⁎
RJKB-T18	+	+	R
RJKB-T19	+	+	R
RJKB-T20	+	+	R
RJKB-T22	−	−	S⁎
RJKB-T24	−	−	S⁎

+, Amplification of PCR product; −, No amplification of PCR product

R, resistance; S, susceptible.

⁎ Fusarium yellows resistance has previously been examined by inoculation test [3].

**Table 2:** Genotype of alleles of clubroot disease.

	Marker		Estimation of	Marker	Estimation of	Marker	Estimation of *Crr3* allele
Lines	CRaim-T	craim-Q	*CRa* allele	mCrr1a	*Crr1a* allele	OPC11-2S
RJKB-T01	−	+	S	−	nd	SB	S
RJKB-T02	−	+	S	LB	S	SB	S
RJKB-T03	−	−	nd	SB	R	SB	S
RJKB-T04	−	+	S	LB	S	SB	S
RJKB-T05	+	−	R	LB	S	SB	S
RJKB-T06	−	−	nd	LB	S	SB	S
RJKB-T07	+	−	R	−	nd	SB	S
RJKB-T08	−	−	nd	SB	R	SB	S
RJKB-T09	−	−	nd	−	nd	SB	S
RJKB-T10	−	+	S	LB	S	SB	S
RJKB-T11	−	−	nd	LB	S	SB	S
RJKB-T12	+	−	R	LB	S	SB	S
RJKB-T13	+	−	R	LB	S	SB	S
RJKB-T14	+	−	R	−	nd	SB	S
RJKB-T15	−	+	S	LB	S	SB	S
RJKB-T16	−	+	S	LB	S	LB	R
RJKB-T17	−	+	S	LB	S	SB	S
RJKB-T18	+	−	R	LB	S	SB	S
RJKB-T19	+	−	R	LB	S	SB	S
RJKB-T20	+	−	R	LB	S	SB	S
RJKB-T22	+	−	R	SB	R	SB	S
RJKB-T24	−	+	S	SB	R	SB	S


Marker	Estimation of	Marker	Estimation of	Marker	Marker	Estimation of *CRc* allele
TCR108	*CRb*^*Zhang*^ allele	B0902	*CRb*^*Kato*^ allele	B50-C9	B50-6R
+	R	LB	S	−	+	S
−	S	LB	S	−	+	S
+	R	LB	S	−	+	S
+	R	LB	S	−	+	S
+	R	SB	R	−	+	S
+	R	LB	S	−	+	S
+	R	SB	R	−	+	S
+	R	LB	S	−	+	S
+	R	LB	S	−	−	nd
−	S	LB	S	−	+	S
+	R	LB	S	−	+	S
+	R	SB	R	−	+	S
+	R	SB	R	−	+	S
−	S	SB	R	−	−	nd
−	S	LB	S	−	+	S
−	S	LB	S	−	+	S
+	R	LB	S	−	+	S
+	R	SB	R	−	+	S
+	R	SB	R	−	+	S
+	R	SB	R	−	+	S
−	S	SB	R	−	+	S
−	S	LB	S	−	+	S

+, Amplification of PCR product; −, No amplification of PCR product

R, resistance; S, susceptible

LB, Larger band; SB, smaller band

nd, clubroot disease resistance cannot be expected because of absence of PCR product.

**Table 3:** *S* haplotype and strength of self-incompatibility.

Lines	*S* haplotype	Number of seeds per flower⁎
RJKB-T01	*S54*	0.85±0.42
RJKB-T02	*S60*	1.59±0.31
RJKB-T03	*S60*	2.46±0.27
RJKB-T04	*S40*	0.10±0.04
RJKB-T05	*S40*	nd
RJKB-T06	*S60*	nd
RJKB-T07	*S40*	3.02±0.51
RJKB-T08	*S60*	2.03±0.28
RJKB-T09	*S40*	1.45±0.74
RJKB-T10	*S40*	0.00±0.00
RJKB-T11	*S54*	0.27±0.12
RJKB-T12	*S99*	0.09±0.03
RJKB-T13	*S40*	1.10±0.24
RJKB-T14	*S40*	0.59±0.23
RJKB-T15	*S40*	1.03±0.27
RJKB-T16	*S40*	0.14±0.07
RJKB-T17	*S54*	0.57±0.17
RJKB-T18	*S99*	0.43±0.13
RJKB-T19	*S99*	0.49±0.29
RJKB-T20	*S25*	0.76±0.12
RJKB-T22	*S46*	nd
RJKB-T24	*S40*	nd

nd represents׳׳no data׳׳.

⁎ Lower values for the number of seeds per flower indicate higher strength of self-incompatibility.

**Table 4:** Sequences of DNA markers.

Name	Primer sequences (5′-3′)		Target
**Fusarium yellows**			
Bra012688m-F/R	AGTCGCTTGGAAGTCTGAGG	GAGCTAACCAACTATACATTGAACC	Bra012688
Bra012689m-F/R	GCATCAAGGCAAAAATGTCA	CATTATAGTAGAACCCAAGTTGATCC	Bra012689

**Clubroot disease**			
CRaim-T-FW/RV	TATATTAATGATAAAGCAGAAGAAGAAA	AATGCGACTGAGAAAGTTGTAG	*CRa*
craim-Q-FW1/RV	TGAAGAATGCGGGCTACGTCCTCTGAAATC	GAAGTAGATGAACGTGTTTATTTTAGAAA
OPC11-2S-F/R	GTAACTTGGTACAGAACAGCATAG	ACTTGTCTAATGAATGATGATGG	*Crr3*
mCrr1a-F/R	CGATGACATGTCTGCCTTCT	TCTGAGATTCAACCGCTTCA	*Crr1a*
TCR108-F/R	CGGATATTCGATCTGTGTTCA	AAAATGTATGTGTTTATGTGTTTCTGG	*CRb*^*Zhang*^
B0902-F/R	AGCCTTGCGTAAAAGCAACTAC	GTTTGGAATCCGACAAATACATCCAT	*CRb*^*Kato*^
B50-C9-FW/B50-RV	GATTCAATGCATTTCTCTCGAT	CGTATTATATCTCTTTCTCCATCCC	*CRc*
B50-6R-FW/B50-RV	AATGCATTTTCGCTCAACC	CGTATTATATCTCTTTCTCCATCCC

***S*****haplotype**			
PS5/PS15	ATGAAAGGCGTAAGAAAAACCTA	CCGTGTTTTATTTTAAGAGAAAGAGCT	Class-I *SLG*
PS3/PS21	ATGAAAGGGGTACAGAACAT	CTCAAGTCCCACTGCTGCGG	Class-II *SLG*
